# Neonatal Electroencephalogram Recording with a Dry Electrode Cap: A Feasibility Study

**DOI:** 10.3390/s25030966

**Published:** 2025-02-05

**Authors:** Amirreza Asayesh, Indhika Fauzhan Warsito, Jens Haueisen, Patrique Fiedler, Sampsa Vanhatalo

**Affiliations:** 1 BABA Center, Pediatric Research Center, Departments of Physiology and Clinical Neurophysiology, New Children’s Hospital and HUS Imaging, Helsinki University Hospital and University of Helsinki, 00014 Helsinki, Finland; amirreza.asayesh@helsinki.fi; 2 Institute of Biomedical Engineering and Informatics, Technische Universität Ilmenau, 98693 Ilmenau, Germany; indhika-fauzhan.warsito@tu-ilmenau.de (I.F.W.);; 3 Biomagnetic Center, Department of Neurology, University Hospital Jena, 07747 Jena, Germany

**Keywords:** neonatal EEG, dry electrode cap, gel-based cap, feasibility study

## Abstract

This study investigates the feasibility of a dry electrode cap design for neonatal electroencephalogram (EEG) recordings. Recordings on a phantom and a real infant are compared between a novel dry electrode cap and a clinically used gel-based electrode cap. The phantom recordings included measuring both the electrode contact force and the signal quality during still and respiration-like head motion. The real infant recordings were assessed for the EEG signals’ spectral characteristics, including powerline interference. Compared to gel-based caps, the dry caps showed a largely comparable skin force, an expectedly greater sensitivity to motion-induced artifacts, and a slightly lower powerline interference. Recordings on the real infant showed no significant skin marks after using the dry electrode, and the spectral compositions were comparable between dry- and gel-based electrode caps. These findings suggest that neonatal EEG recordings with a dry electrode cap are technically feasible, but movement-related artifacts, such as respiration in a supine lying infant, may challenge long-term recordings of spontaneous EEG activity. Yet, the ease of use of dry electrode caps calls for future studies to define the optimal use case in neonatal recordings.

## 1. Introduction

Scalp-recorded electroencephalography (EEG) is routine in neonatal medicine and other newborn studies [1,2], and several types of EEG electrodes have been introduced to the market for home health monitoring, clinical use, and research applications [3]. Electrode–skin contact is typically created using electrolyte gel or paste and is, therefore, called a gel-based electrode or cap [4] https://www.babacenter.fi/nemo/index.html, accessed on 2 February 2025. However, using such electrodes typically requires some skin preparation, the application of conductive gels or pastes, and an extended cleanup time after the recording. Gel-based electrodes, such as those used in neonatal EEG recordings, present several practical challenges [4,5,6,7,8]: their application needs substantial expertise, especially due to time-consuming skin abrasions and accurate gel application. Gel-based electrodes also need frequent maintenance due to the drying of the gel, which degrades EEG signals over hours. The gel-based electrodes, particularly those integrated into EEG caps, need a substantial effort to clean them after the EEG recording, while the gel itself needs to be removed from the scalp and hair [9,10,11,12]. As an alternative, dry electrodes were proposed in the 1990s to minimize skin preparation and make setup and cleaning faster and easier compared to gel-based electrodes [13,14,15]. The dry electrodes consist of an inert conductive material of varying shapes [16], and it is electrically coupled to the skin with mechanical force.

A persistent challenge with the dry electrode design is striking a balance between sufficient electrical contact to provide high-quality signals without causing an excessively uncomfortable mechanical force on the scalp. A flat, dry electrode design would be softer and more flexible, but it would require a hair-free scalp or substantial skin preparation or prepared skin to maintain adequate contact [17,18]. Recently introduced bristle electrodes with softer pins were found to provide sufficient skin contact with comfortable wear in adults [19,20]. Recent studies evaluating the performance of dry EEG electrodes have often reported increased noise levels compared to gel-based electrodes [20,21]. Yet, further studies have demonstrated the reproducibility of results across multi-center evaluations and their potential utility in many clinical and research applications [15].

The balance between mechanical force and wearing comfort is particularly relevant for neonatal applications, where achieving optimal contact without compromising skin integrity is a major challenge [22,23,24]. Since infants’ skin is significantly more sensitive than adult skin, developing soft electrodes with less mechanical stress has been critical [25,26], and the current dry electrode designs for neonates still struggle with variable skin contact, i.e., impedance and high susceptibility to movement artifacts [27].

The development of neonatal dry electrodes is further complicated by the difficulties in their evaluation due to regulatory and ethical considerations in vulnerable populations [28,29]. A partial solution to these challenges is to carry out preclinical testing on a phantom head that would simulate the key anatomical and electrical properties of neonatal scalp placement under controlled conditions. Such testing would allow for iterative improvements while mitigating risks to neonates [30].

This study aims to test the feasibility of a novel dry electrode cap design using a combination of EEG and force recordings on a phantom head and EEG recordings on a real newborn infant. The key study questions are as follows: (i) Are the mechanical contact forces between electrodes and the infant scalp within tolerable limits? (ii) What is the signal quality in the dry electrode recordings, as measured by powerline interference and movement-related noise? Such a combination of preclinical (phantom) and clinical (real infant) recordings would inform the future selection of use cases for dry electrode EEG recordings in newborns and other young infants.

## 2. Materials and Methods

### 2.1. Overview

**Figure 1:**
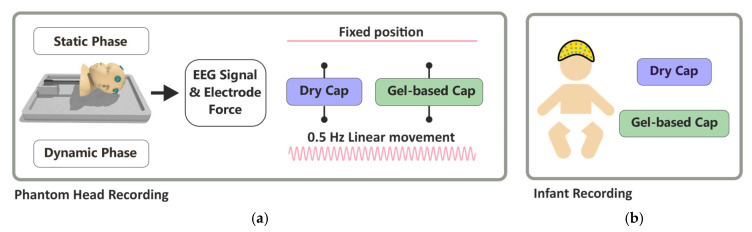
Recording protocols. Phantom recordings: (**a**) the included measurement of both the EEG signal and the contact force, using both a dry- and a gel-based electrode cap, respectively. Recording conditions were either with the head lying still (“fixed position”) or the head mechanically moving to mimic respiration at 30 breaths per minute. (**b**) The real infant recording included dry- and gel-based electrode caps, one after the other, during a natural sleep. Signal analyses were identical in all recordings.

Figure 1 presents an overview of the study design. Recordings were performed using a phantom head (left) and a real infant (right), and we compared recordings with a dry electrode cap and a gel-based electrode cap in identical settings. Phantom recordings included a static phase (no movement) and a movement phase (respiration-like movement).

### 2.2. EEG Caps and EEG Recordings

**Figure 2:**
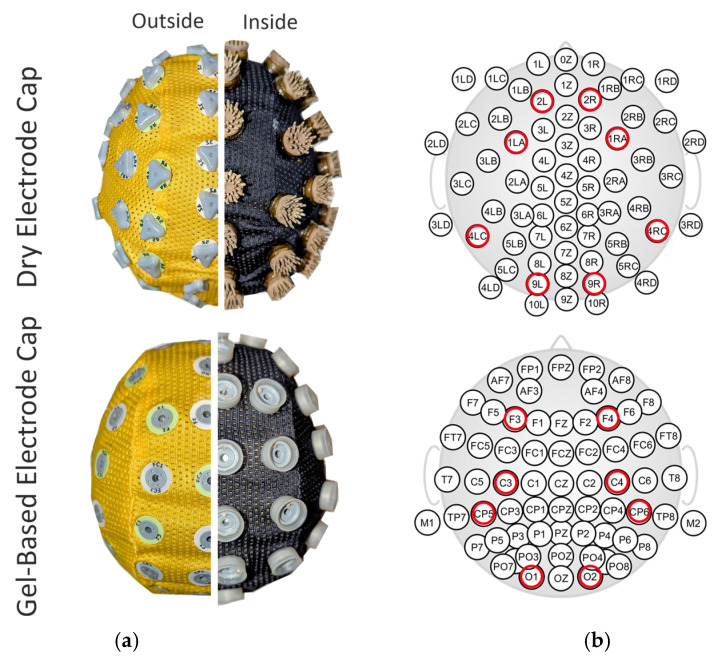
Comparison of the 64-channel dry electrode: (**a**) dry electrode and gel-based electrode caps; (**b**) montages. The photographs show the outside and inside view of the caps. The electrode layout shows the placement of the force sensors (red circles) under the given electrodes.

EEG recordings were conducted using a referential 64-channel amplifier (eego, ANT Neuro B.V., Hengelo, The Netherlands). Both the dry- and gel-based electrode caps used the same fabric and other materials, except for the electrode element (see also Figure 2). The infant-size dry electrode cap was constructed using silver/silver chloride (Ag/AgCl)-coated polyurethane (PU) multipin electrodes (waveguard touch, shore hardness A60, ANT Neuro B.V.), as previously used for adult-size dry electrode caps [25]. Ground and reference electrodes were positioned at the mastoids M1 and M2, respectively, on the phantom head. For the neonate, they were placed on the shoulder and neck, respectively, using self-adhesive hydrogel electrodes and no skin rubbing. The recordings with gel-based electrodes were performed with commercially available neonatal size caps (see Figure 2a, lower row) equipped with sintered Ag/AgCl electrodes (waveguard original, ANT Neuro B.V.) [31,32,33,34,35,36], and skin contact was established with an electrolyte gel (Electro-Gel, Electro-Cap International Inc., Eaton, OH, USA) injected into the gel chambers.

### 2.3. Phantom Head Recordings

A 3D-printed head phantom was created using a 3D model from the authentic neonatal magnetic resonance imaging of a healthy infant. The phantom was printed using polylactic acid material with a 40% infill density, resulting in a weight of approximately 600 g.

Force measurements: The electrode contact force was measured with eight force resistance sensors (FSRs), including Interlink FSR-402 (Interlink Electronics, Irvine, CA, USA), placed at the frontal, central, temporal, and occipital scalp locations (see Figure 2) using 3D-printed holders attached to the phantom. The sensors were connected to a pair of ADS1115 analog-to-digital converters communicating with a Raspberry Pi 3B+ single-board computer via the I^2^C protocol and transmitted over an ethernet connection to a custom user interface developed in LabVIEW 2023 on a desktop computer. This interface enabled the real-time visualization of the force readings, supporting our understanding of how head movements cause forces on the scalp. The force values were displayed as Newtons. The electrode force recordings were conducted separately for the dry- and gel-based electrode caps by fixing the eight sensor holders on the phantom with double-sided foam tape. A hydratable and disposable fabric layer was placed over the force sensors to serve as a conductive medium for saline-saturated EEG electrodes.

Recording session: Force sensor calibration was performed at the beginning of each recording session. The FRS measurements were calibrated using seven weights, ranging from 50 g to 500 g, which was considered to cover the full range of expected measures. The measured FRS data were then fitted using the Cholesky decomposition algorithm and 4th-order polynomial function. The reliability of the calibration curve was confirmed by repeatedly applying the weights in a random order to exclude biases and/or noise between the measurements. Each recording session began with the new placement of the cap over the phantom. To ensure real-life relevant cap placement, the most challenging part of the infant EEG study, we employed an EEG technician with over 10 years of experience in performing clinical infant EEG recordings. For the static phase recordings (first 5 min), the base of the phantom head was securely attached to a linear motor placed on a mattress to ensure stability and prevent unwanted movement. For the dynamic phase (5 min), the phantom head was moved linearly by 5 mm at a rate of 30 cycles per minute, which corresponds to a typical infant’s respiration rate. There is clearly some variability between subjects and, over time, in the magnitude of respiration movements. Here, we chose 5 mm as a safe upper-range measure to reflect significant and visually clear head movements. The respiration rate (30/min) is a typical respiratory frequency in human infants, with a developmental decline from ~40/min to nearly 20/min during the first year of life [37]. These recording sessions (5 + 5 min) were repeated five times for both cap types.

### 2.4. Infant EEG Recording

EEG signals were recorded from a one-month-old newborn infant with a head circumference of 34.5 cm using both dry- and gel-based 64-channel electrode caps. Each recording lasted approximately 15 min. In both sessions, the infant was in a sleeping state, providing stable conditions for data collection. The dry electrode cap was placed on the infant’s head without any skin preparation. Thereafter, the gel-based electrode cap was applied using electrolyte gel at each electrode site without skin preparation. The same ground and reference electrode placements were used. After each recording session, observations were made regarding the skin condition upon cap removal, noting any skin marks or redness to assess the tolerability of each cap type.

### 2.5. Data Analysis

#### 2.5.1. Preprocessing

**Figure A1:**
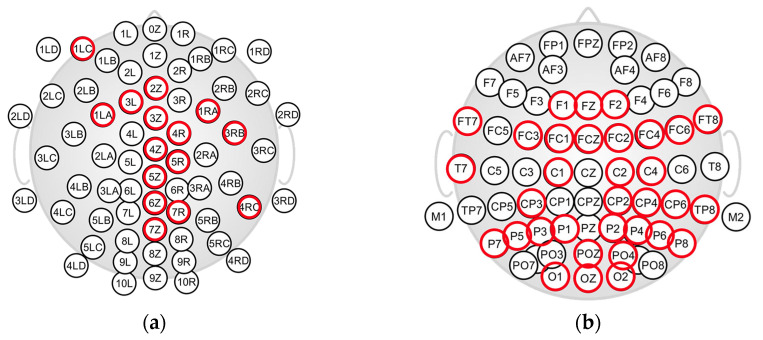
Removed channels from (**a**) the dry cap. The 4RD electrode was removed from dry electrode cap channels while being worn; (**b**) removed channels from the gel-based cap.

All EEG signal analyses were identical for both the phantom head and infant data, as well as for both dry- and gel-based electrode caps. EEG channels with flat signals (zero variance) were excluded from phantom head recordings. In real infant recordings, channels were excluded both when showing zero variance, when exhibiting motion artifacts, or inadequate skin–electrode contact (Figure A1). Subsequently, the data were filtered between 0.2 and 35 Hz (6th-order IIR band-pass filter). Also, a 50 Hz notch filter was applied to mitigate powerline interference, except for the quantitation of powerline interference (RMS at 50 Hz). The EEG data from the real infant recordings were filtered using the clinically customary range 1–30 Hz and an additional 50 Hz notch filter. Additional wavelet denoising (Daubechies, level 4) was applied to reduce electrocardiography artifacts. The data were then converted to a bipolar montage (Table A1) before computational assessment. The force signals were visually inspected for apparent noise and artifacts, and a 24 Hz low-pass filter was applied to remove any high-frequency artifacts.

#### 2.5.2. Force Measurements

The first 5 s of data, typically contaminated by initial artifacts, were trimmed, and force values below 0.2 N were set to 0. Root mean square (RMS) values with standard deviation and peak force values were calculated. Later, these values were scaled in relation to their minimum and maximum values for compatibility. Force differences between static and dynamic phases were assessed across five recording sessions.

#### 2.5.3. EEG Signal Analysis

In the phantom recordings, a total of 100 epochs were analyzed, with 25 epochs allocated to each condition: dry static, dry dynamic, gel-based static, and gel-based dynamic conditions. RMS values at 0.5 Hz were calculated to quantify the artifacts from respiration-like movements. RMS power at 50 Hz was estimated to assess powerline interference.

We chose to use the frequency band terminology that is commonly adopted in the EEG literature. Since the exact band limits are somewhat variable between studies, we also defined them numerically in our work for transparency. Most of the neonatal EEG phenomena are by nature multi-frequency phenomena, and they include one or more of the studied frequency bands, except for the higher (gamma) frequencies [1,2], which do not exist in substantial amounts in neonatal EEGs; hence, they were removed by low-pass filtering in our analyses. In the infant recordings, power spectral densities (PSDs) were computed for both dry- and gel-based caps across key frequency bands (delta: 1–4 Hz, theta: 4–8 Hz, alpha: 8–13 Hz, beta: 13–30 Hz) using Welch’s method. Later, RMS values were calculated in the delta band (1.5–4 Hz) to evaluate the signal quality in low-frequency ranges.

### 2.6. Statistical Analysis

The Shapiro–Wilk test indicated non-normal data distributions; as a result, we chose to use non-parametric comparisons with the Mann–Whitney U test for comparisons of median values between static and dynamic phases for both caps. This comparison was performed for the force values, the RMS estimates, and the PSD estimates. False Discovery Rate (FDR) correction was applied to control for multiple comparisons. The significance threshold was set at *p* ≤ 0.05.

## 3. Results

### 3.1. Phantom Head Recording, Force

**Figure 3:**
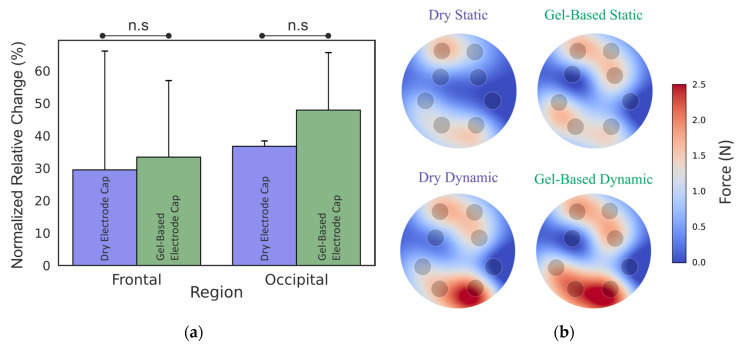
Electrode contact forces: (**a**) change in electrode contact force (RMS) between the static and dynamic phase; (**b**) peak forces of the electrodes in dry- and gel-based caps in the static phase and dynamic phase.

Figure 3 represents the normalized relative RMS change in the dynamic-to-static phase for dry- and gel-based caps in frontal and occipital regions. And the peak force for each sensor in the static and dynamic state was determined for dry electrode caps and gel-based caps. There were no significant differences between the electrode contact forces between dry- and gel-based caps.

Head movement at respiration rate caused an average increase of 29.5% (frontal) to 33.4% (occipital) in force in the dry electrode caps compared to the static situation. In the gel-based cap, the corresponding force increase with respiration-like movements was 36.7% in the frontal region and 47.9% in the occipital region. The peak (maximum) force was 1.67 for the dry cap and 1.65 for the gel-based cap. The peak force for the dry cap in the dynamic phase was 2.57 N and 2.57 for the gel-based cap.

### 3.2. Phantom Head Recording, EEG Signal

**Figure 4:**
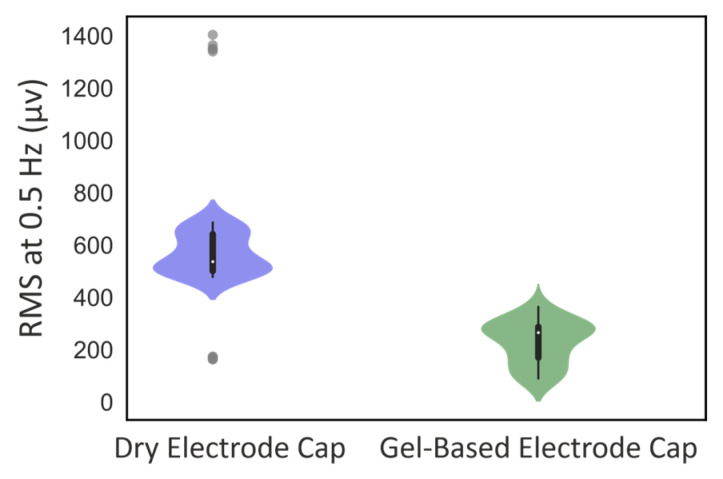
Violin plot of the magnitude of respiration-like movement artifacts (RMS at 0.5 Hz).

Figure 4 shows the EEG effect of respiration-like movements for the RMS at 0.5 Hz. The mean respiration-like movement artifact was significantly higher in the dry electrode cap compared to the gel-based electrode cap (RMS at 0.5 Hz 576 vs. 228 µv, respectively; *p* < 0.001). This indicates that the dry electrode cap is more susceptible to respiration-like movements compared to gel-based electrode caps.

**Figure 5:**
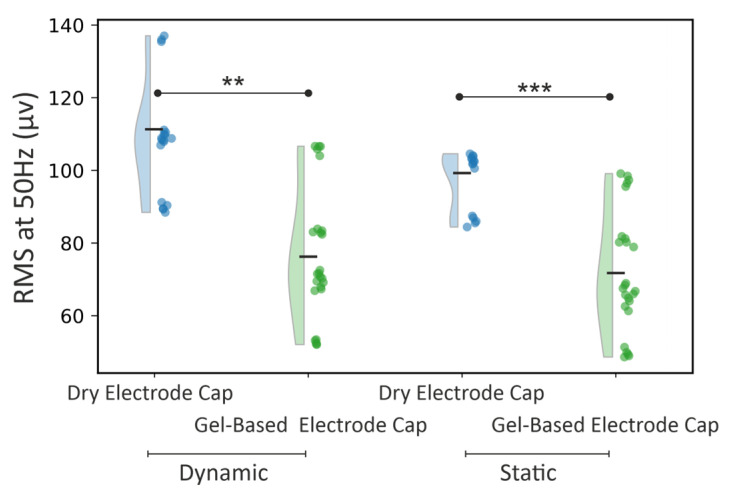
Comparison of the powerline interference (RMS at 50 Hz) between dry- and gel-based electrode caps in different recording conditions. Distributions are shown as violin plots from the measured 25 epochs that are shown individually on the left side of each violin plot. The median of the distribution is depicted with a black horizontal line. The significance level for the comparisons is shown with asterisks: ** (*p* < 0.01), *** (*p* < 0.001).

As shown in Figure 5, there is also a significant difference between dry- and gel-based electrode caps in terms of powerline interference (RMS at 50 Hz). In the static condition, the dry cap exhibited an average RMS of 99.30 µV compared to 71.78 µV for the gel-based cap (*p* < 0.05). In the dynamic condition, this difference was even more pronounced, with the dry cap measuring 111.34 µV versus 76.28 µV for the gel-based cap (*p* < 0.01).

### 3.3. Real Infant Signal Analysis

**Figure 6:**
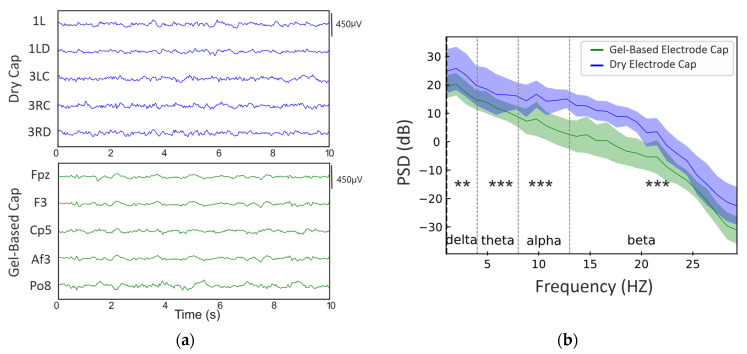
Comparison of dry- and gel-based electrode cap recordings from a real human infant: (**a**) ten-second examples of EEG signals using dry- (above) and gel-based (below) electrode caps, with signals shown after clinically used 0.5–35 Hz filtering to remove most of the powerline noise; (**b**) power spectrum density plots with standard deviations for all 15 EEG epochs analyzed, and statistical comparison between the gel-based cap and dry electrode cap at the canonical delta, theta, alpha, and beta frequencies. Statistical significance is shown with asterisks: ** (*p* < 0.01); *** (*p* < 0.001).

In the visual review, the EEG recordings from the dry- and gel-based electrode caps appeared largely comparable when mechanically stable time epochs were compared. Figure 6 shows examples of the raw EEG signal at comparable frontal, central, and occipital scalp locations.

**Figure 7:**
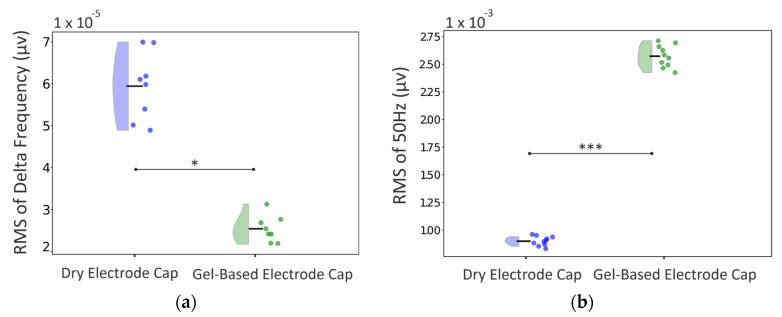
Frequency-specific differences between dry- and gel-based electrode caps: (**a**) for respiration-related low frequency and (**b**) powerline-related 50 Hz frequency. Distributions are shown as violin plots from the 15 epochs measured that are shown individually on the left side of each violin plot. The median of the distribution is depicted with a black horizontal line. Asterisks indicate the significance levels: * (*p* < 0.05); *** (*p* < 0.001).

A comparison of the 1–30 Hz power spectra between dry- and gel-based electrode EEG caps is shown in Figure 7 The overall spectral shape was comparable between the caps, while the median power was consistently somewhat higher in dry electrode caps, with the following significant difference by frequency bands: delta (*p* ≤ 5 × 10⁻^3^), theta (*p* ≤ 1 × 10⁻⁴), alpha (*p* ≤ 1 × 10⁻⁴), and beta (*p* ≤ 1 × 10⁻⁴). These differences were also significant after the FDR correction.

Moreover, there were frequency-specific differences (Figure 7). The median delta frequency RMS was significantly higher in the dry electrode caps (60 vs. 25 µv, respectively; *p* < 0.02). The powerline interference (RMS at 50 Hz), in turn, was significantly higher in the gel-based electrode caps (gel-based: 2075.0 vs. 900 µv; *p* < 0.001).

### 3.4. Practical Observations of Using the Dry Electrode Cap

The application of the dry electrode cap was clearly faster compared to the gel-based electrode cap. Placing the cap on the scalp was comparable, but the time-saving qualities came from no need to apply gel, which typically takes most of the application time. The dry electrode cap was also faster to remove after the end of the recording because there was no need to remove the electrode gel (used with the gel-based cap) from the infant’s scalp or the electrode holders (see Figure 2), which typically takes several minutes of manual labor. A comparison of skin markings showed no practical difference. Some transient, lightly reddish skin indentations were seen immediately after the recording, but they disappeared within an hour, and they caused no apparent discomfort. Comparable markings are routinely seen with gel-based electrode caps after neonatal EEG recordings, as the silicone-based rims of the gel chamber press against the scalp, most typically below the hairline on the forehead and temporal regions.

## 4. Discussion

The present findings demonstrate the feasibility and highlight some challenges associated with using a dry electrode cap for EEG recordings in human infants, especially near newborn age. The findings on force distributions and signal quality are generally in line with prior studies using dry electrode caps in adults [19]. Here, we extend the prior literature by showing that the same dry electrode design can also be adopted in a newborn head dimension with a significantly smaller diameter, hence a much smaller cap surface area. We also extend the prior literature by combining preclinical testing with phantom-to-clinical testing on a real human infant. In theory, this could have caused practical issues in terms of a too-high contact force or, likewise, too-low signal quality.

### 4.1. Force Distributions

The recorded force differences between the dry- and gel-based caps in static and dynamic phases were found to be comparable, though slight variations were observed between scalp regions. Of clinical importance in the context of infant recordings, we did not observe any skin abrasions or other clinically meaningful skin marks to indicate potential skin damage as a result of the observed contact forces; the higher force recorded in the occipital region (2.57 N peak force) for the gel-based cap can likely be attributed to its vertically aligned electrode configuration, which concentrates the force on a single point. The horizontal alignment of the electrode positions in the dry cap aimed to reduce contact force with apparent success (2.57 N in comparison to 4 N in adults [19]). However, there was a clear increase in the contact force during respiration-like movements, suggesting a need for further refinement in the electrode and/or cap design. [18]. The increase in contact force during respiration-like movements may be attributed to the relatively rigid nature of the given prototype. This rigidity is caused by three main factors: (i) the number and density of the electrodes; (ii) the current mechanical fixation of the electrodes within the textile; and (iii) the flexibility of the textile. Consequently, further research and development should focus on improving both the fixation mechanism of the dry electrodes and the chosen textile to increase flexibility during movements and, thus, reduce the effect of movements on the resulting contact force.

### 4.2. EEG Signal Quality

Measures of signal quality indicated notable differences between the dry- and gel-based electrode caps. In the phantom recordings, the dry electrode caps showed a higher sensitivity to respiration movements that were well controlled by mechanical movement and, hence, isolated from any spontaneous low-frequency EEG activity [33,34,35,38,39] that would confound the artifact estimation in the real infant recordings. The real infant recordings showed comparable PSD shapes; however, the dry electrode cap showed significantly higher RMS values at the low (delta) frequency band, which is consistent with respiration-related movement artifacts. These findings together indicate that the dry electrode cap is more susceptible to motion artifacts; however, it can record reasonably well at higher EEG signal frequencies. Future development would benefit from efforts to optimize the mechanical design in dry electrode caps, with emphasis on the electrical properties during slow movements. Another limitation is the sequential setup, where one cap is placed at a time. Parallel setups can overcome this limitation but require special caps [40]. Future work should include additional analyses restricted to common electrode positions to ensure the comparability of slow-wave activity between dry- and gel-based systems. This approach would help to clarify whether the observed differences arise from electrode-specific properties or artifact-prone regions of the scalp.

### 4.3. Practical Implications for Neonatal Applications

In newborn and other infant EEG applications, practicalities such as ease of use, short setup times, and infant comfort are critical because the possible recording time window with steady sleep is very brief in newborn infants. The dry electrode caps perform well in these terms. However, the increased sensitivity to motion and powerline noise in the dry electrode caps presents a trade-off that needs to be addressed before clinical adoption. Future iterations of the dry cap could incorporate motion-canceling algorithms or mechanical adjustments to improve signal stability during movement. The respiration-like movements are obviously not concerning in adult recordings; however, infant recordings are primarily performed during sleep when the infant’s head is lying against a pillow [31,34,38,41]. Therefore, head movement artifacts may preclude the analysis of spontaneous activity, at least at lower frequencies, while the present cap design could already be utilized to analyze brief segments, such as evoked and event-related potentials. While the infant sample recordings provide critical insights into real-world performance, the small sample size limits the generalizability of the findings. Further studies with larger cohorts are necessary to validate the dry cap’s efficacy in diverse clinical scenarios.

## 5. Conclusions

The dry electrode cap design used in this study shows promise as a viable alternative to traditional gel-based electrodes for newborn and other infant EEG recordings. While the findings support practical utility and potential benefits in performing the recording, there are challenges related to the signal quality, such as respiration-related motion artifacts or powerline interference. Future work using a combination of phantom and real-world infant recordings could provide an optimal testbed to iterate the electrode and cap designs. Further studies with larger cohorts are necessary to validate the ultimate use cases, including clinical utility, of the dry cap solution in the studies of human infants.

## Data Availability

The EEG dataset presented in this article is legally considered patient data and cannot be made freely available without relevant research contracts with the owner of the patient data (Helsinki University Hospital, Helsinki, Finland).

## Appendix A

Table A1: Bipolar montage including the dry cap equidistant and 10–20 gel-based cap electrode list in parenthesis.

[3Z(Fp2)-7Z(AF8), 7Z(AF8)-1RB(F8), 1RB(F8)-2LA(FT8), 2LA(FT8)-2RB(T8), 2RB(T8)-4LC(TP8), 4LC(TP8)-3RC(P8), 3RC(P8)-4LB(PO8), 4LB(PO8)-3RD(O2), 1Z(Fp1)-3LD(AF7), 3LD(AF7)-4Z(F7), 4Z(F7)-3RA(FT7), 3RA(FT7)-4R(T7), 4R(T7)-2RA(TP7), 2RA(TP7)-2RC(P7), 2RC(P7)-4RC(PO7), 4RC(PO7)-2RD(O1), 3Z(Fp2)-10R(F6), 10R(F6)-3R(Fc6), 3R(Fc6)-6L(C6), 6L(C6)-2LC(CP6), 2LC(CP6)-5LC(P6), 5LC(P6)-3LA(PO6), 3LA(PO6)-3RD(O2), 1Z(Fp1)-6Z(F5), 6Z(F5)-2L(Fc5), 2L(Fc5)-8R(C5), 8R(C5)-1RA(Cp5), 1RA(Cp5)-5R(P5), 1RA(Cp5)-5RC(PO5), 5RC(PO5)-2RD(O1), 3Z(Fp2)-8Z(AF4), 8Z(AF4)-1LB(F4), 1LB(F4)-8L(FC4), 8L(FC4)-2LB(C4), 2LB(C4)-5L(CP4), 5L(CP4)-3LC(P4), 3LC(P4)-5RB(PO4), 5RB(PO4)-3RD(O2), 1Z(Fp1)-9Z(AF3), 9Z(AF3)-1L(F3), 1L(F3)-9L(FC3), 9L(FC3)-1LC(C3), 1LC(C3)-6R(CP3), 6R(CP3)-3LB(P3), 3LB(P3)-5LB(PO3), 5LB(PO3)-2RD(O1), 3Z(Fp2)-10L(F2), 10L(F2)-3L(Fc2), 3L(Fc2)-7R(C2), 7R(C2)-1RD(CP2), 1RD(CP2)-4RD(P2), 4RD(P2)-3RD(O2), 1Z(Fp1)-5Z(F1), 5Z(F1)-2R(Fc1), 2R(Fc1)-7L(C1), 7L(C1)-1LD(CP1), 1LD(CP1)-4LD(P1), 4LD(P1)-2RD(O1), 2Z(Fpz)-1R(Fz), 1R(Fz)-9R(FCz), 9R(FCz)-1RC(Cz), 1RC(Cz)-9Z(AF3), 3RB(Pz)-2LD(POz), 2LD(POz)-4RB(Oz)]

Figure A1: Removed channels in the signal analysis phase considered as bad channels.

**Table A1:** Table of terms.

Acronym/Term	Full Name
EEG	Electroencephalogram
FSR	Force Sensing Resistor
Ag/AgCl	Silver/Silver Chloride
RMS	Root Mean Square
PSD	Power Spectral Density
FDR	False Discovery Rate
I^2^C	Inter-Integrated Circuit
Powerline Interference	Electrical Noise at 50 Hz
